# Post hoc analyses of the impact of previous medication on the efficacy of lisdexamfetamine dimesylate in the treatment of attention-deficit/hyperactivity disorder in a randomized, controlled trial

**DOI:** 10.2147/NDT.S68273

**Published:** 2014-10-29

**Authors:** David R Coghill, Tobias Banaschewski, Michel Lecendreux, César Soutullo, Alessandro Zuddas, Ben Adeyi, Shaw Sorooshian

**Affiliations:** 1 Division of Neuroscience, University of Dundee, Dundee, UK; 2 Child and Adolescent Psychiatry and Psychotherapy, Central Institute of Mental Health, Medical Faculty Mannheim, University of Heidelberg, Mannheim, Germany; 3 Paediatric Sleep Centre and National Reference Centre for Orphan Diseases: Narcolepsy, Idiopathic Hypersomnia and Kleine-Levin Syndrome, Robert-Debré University Hospital, Paris, France; 4 Child and Adolescent Psychiatry Unit, Department of Psychiatry and Medical Psychology, University of Navarra Clinic, Pamplona, Spain; 5 Department of Biomedical Sciences, Section of Neuroscience and Clinical Pharmacology, University of Cagliari, Cagliari, Italy; 6 Shire, Wayne, PA, USA; 7 Shire, Eysins, Switzerland

**Keywords:** attention-deficit/hyperactivity disorder, lisdexamfetamine dimesylate, methylphenidate, central nervous system stimulants

## Abstract

**Background:**

Following the approval of lisdexamfetamine dimesylate (LDX) in several European countries for the treatment of attention-deficit/hyperactivity disorder (ADHD) in children and adolescents with an inadequate response to methylphenidate (MPH) treatment, the aim of the present analysis was to establish the response to LDX in subgroups of patients with different ADHD medication histories.

**Methods:**

This was a post hoc subgroup analysis of data from a 7-week, European, double-blind, dose-optimized, Phase III study. Patients aged 6–17 years were randomized 1:1:1 to LDX, placebo, or osmotic-release oral system methylphenidate (OROS-MPH). OROS-MPH was included as a reference arm rather than as a direct comparator. Efficacy was assessed in patients categorized according to their ADHD medication history using the ADHD Rating Scale IV and Clinical Global Impressions-Improvement (CGI-I) scores.

**Results:**

The difference between active drug and placebo in least-squares mean change from baseline to endpoint in ADHD Rating Scale IV total score (95% confidence interval) was similar between the overall study population (n=317; LDX, −18.6 [−21.5, −15.7]; OROS-MPH, −13.0 [−15.9, −10.2]) and treatment-naïve individuals (n=147; LDX, −15.1 [−19.4, −10.9]; OROS-MPH, −12.7 [−16.8, −8.5]) or patients previously treated with any ADHD medication (n=170; LDX, −21.5 [−25.5, −17.6]; OROS-MPH, −14.2 [−18.1, −10.3]). In addition, similar proportions of patients receiving active treatment were categorized as improved based on CGI-I score (CGI-I of 1 or 2) in the overall study population and among treatment-naïve individuals or patients previously treated with any ADHD medication.

**Conclusion:**

In these post hoc analyses, the response to LDX treatment, and to the reference treatment OROS-MPH, was similar to that observed for the overall study population in subgroups of patients categorized according to whether or not they had previously received ADHD medication.

## Introduction

Attention-deficit/hyperactivity disorder (ADHD) is characterized by persistent symptoms of hyperactivity/impulsivity and/or inattention, and is estimated to affect approximately 5.9%–7.1% of children and adolescents worldwide.^1^,^2^ Previously thought to be limited to childhood, symptoms of ADHD are now believed to persist into adulthood in approximately two thirds of patients.^3^,^4^ ADHD is associated with significant impairments in academic, social, and interpersonal functioning, highlighting the importance of effective therapeutic options.^1^,^5^

Psychostimulants, including methylphenidate (MPH) and amphetamine, are recognized as effective pharmacological treatments for ADHD.^1^,^6^ In Europe, MPH is generally recommended as the first-line medication for ADHD. The prodrug lisdexamfetamine dimesylate (LDX) is the first long-acting amphetamine-based ADHD medication to be approved in Europe, where it is licensed as a second-line therapy in several countries for the treatment of children and adolescents who have experienced a clinically inadequate response to MPH therapy. LDX has been established as an effective and generally well tolerated treatment for children, adolescents, and adults with ADHD in multiple randomized, double-blind, placebo-controlled trials.^7^–^11^ In a pivotal European, Phase III, double-blind, randomized controlled trial in children and adolescents with ADHD (study SPD489-325; ClinicalTrials.gov identifier NCT00763971), LDX significantly improved ADHD symptoms compared with placebo, as assessed by the ADHD Rating Scale IV (ADHD-RS-IV) and Clinical Global Impressions-Improvement (CGI-I).^10^ In addition to the placebo arm, this European study included osmotic-release oral system MPH (OROS-MPH) as an active reference treatment (as opposed to a direct comparator) to provide study validation and contextualize results. Whilst the study was not designed to support a formal statistical comparison of the two active treatments, a subsequent post hoc analysis indicated that LDX was significantly more effective than OROS-MPH at improving ADHD-RS-IV scores (effect size 0.54; *P*<0.001) and that a significantly (*P*<0.05) greater proportion of patients receiving LDX were classified as treatment responders, based on two of the three response criteria examined, than those receiving OROS-MPH.^12^

The present post hoc analyses examined the impact of previous ADHD medication on the efficacy of LDX treatment in SPD489-325. Because of the recent second-line approval of LDX in several European countries, European patients being considered for LDX treatment should have received other ADHD medications, and for most this is likely to be an MPH-based formulation. Therefore, for clinicians in Europe, it is particularly important to establish the efficacy of LDX in patients who have been previously treated with ADHD medication. The aim of this analysis was to establish whether the LDX treatment response for subgroups of patients with different ADHD treatment histories was similar to that of the overall study population. This analysis did not attempt to compare the relative effects of LDX and the reference treatment OROS-MPH within these patient subgroups because the small number of patients involved would limit the robustness of such analyses to support inferential statistics.

## Materials and methods

The design of study SPD489-325 has been described in detail previously.^10^ This randomized, double-blind, parallel-group, dose-optimized, placebo-controlled study was conducted in accordance with current applicable regulations, the International Conference on Harmonisation of Good Clinical Practice, and local ethical and legal requirements. The study protocol was approved by an independent ethics committee/institutional review board and regulatory agency in each center (as appropriate) before study initiation. Each patient’s parent or legal guardian provided written, informed consent, and, when appropriate, assent was obtained from each patient before commencing study-related procedures.

### Study population

This study enrolled male and female children and adolescents (aged 6–17 years) who satisfied the Diagnostic and Statistical Manual of Mental Disorders, Fourth Edition, Text Revision (DSM-IV-TR) criteria for a primary diagnosis of ADHD, and who had a baseline ADHD-RS-IV total score of 28 or higher. Medication history was obtained at baseline. Patients were excluded from the study if they had previously failed to respond, based on the investigators’ judgment, to an adequate course (dose and duration) of OROS-MPH therapy or if their current ADHD medication provided effective control of symptoms with acceptable tolerability. In addition, individuals with documented allergy, hypersensitivity, or intolerance to amphetamines or MPH were excluded. All patients attended an initial screening visit which included documentation of their lifetime history of therapies for ADHD; use of any investigational compound, clonidine, antipsychotic, anxiolytic, sedative-hypnotic or antidepressant medication within 30 days prior to screening excluded a patient from the study. The use of herbal preparations or melatonin was prohibited during the study. Continued participation in behavioral therapy was permitted, provided that the patient had been receiving the therapy for at least one month at the time of the baseline visit. With the exception of oppositional defiant disorder, this study also excluded patients having a comorbid psychiatric diagnosis with significant symptoms and individuals with a conduct disorder.

### Study design

Following the initial screening visit (visit 1), participants completed a washout period (3–42 days); for all previous medications, the washout period was a minimum of five times the half-life of the medication. Eligible patients were then randomized (1:1:1) at baseline (visit 0) to receive once-daily LDX (30 mg, 50 mg, or 70 mg), placebo, or OROS-MPH (18 mg, 36 mg, or 54 mg) for a 7-week, double-blind, dose-optimized evaluation period (visits 1–7). Investigators were not blinded to patients’ treatment history. The evaluation period was immediately followed by a one-week washout and a follow-up visit (visit 8). Endpoint was defined as the last on-therapy, post-randomization visit at which a valid efficacy score was obtained.

### Efficacy assessments

The primary efficacy outcome in study SPD489-325 was the change in ADHD-RS-IV total score from baseline to endpoint.^13^ This assessment scale consists of 18 items designed to reflect current ADHD symptoms and the total score ranges from 0 to 54. ADHD-RS-IV assessments were performed at baseline (visit 0) and all subsequent treatment visits by a physician experienced in the evaluation of children and adolescents with ADHD. A decrease from baseline in ADHD-RS-IV score indicates improvement in ADHD symptoms.

The key secondary efficacy outcome utilized the CGI scale,^14^ which permits a global evaluation of the patient’s severity of illness and improvement over time. CGI-I was assessed at all post-baseline visits to rate the patient’s improvement from baseline using a 7-point scale ranging from 1 (very much improved) to 7 (very much worse). Patients were categorized as “improved” (defined as a CGI-I score of 1 [very much improved] or 2 [much improved]) or “not improved” (all other scores), and results were expressed as the percentage of improved patients at endpoint. The CGI assessments were completed by a physician experienced in the evaluation of children and adolescents with ADHD.

In these post hoc analyses, changes in ADHD-RS-IV total scores and in the percentage of patients categorized as “improved” on the basis of CGI-I scores were assessed for patients dichotomized according to previous treatment with ADHD medication (treatment-naïve or previously treated). In addition, changes in ADHD-RS-IV total scores were examined specifically in patients who had reported that they had previously received MPH treatment at any time, and in those who had reported receiving MPH treatment in the period immediately prior to randomization in SPD489-325 (defined as having received MPH treatment at any point during the 30 days prior to randomization).

### Statistical analysis

The safety population comprised all patients who were randomized and received at least one dose of investigational product. The full analysis set was defined as all patients who were randomized and received at least one dose of investigational product but excluded 15 patients from one site owing to violations of good clinical practice. Dosing information and efficacy outcomes were assessed in the full analysis set. Statistical analyses included the calculation of least-squares means, which were based on type III sum of squares from an analysis of covariance model for the change from baseline in ADHD-RS-IV total score, including treatment, country, and age groups as fixed effects and baseline value as a covariate. The precision of results and the similarities between subgroups were estimated based on 95% confidence intervals. Effect sizes were calculated as the difference between the least-squares mean scores for the active treatment and placebo groups, divided by the root mean square error obtained from the analysis of covariance model. Effect sizes of 0.2, 0.5, and 0.8 correspond to small, medium, and large magnitudes of effect, respectively.^15^ OROS-MPH was included in SPD489-325 as an active reference arm in order to provide internal validation of the study design. However, SPD489-325 was not designed or powered to support a formal statistical comparison between LDX and OROS-MPH or between treatment-naïve patients and those who had previously received ADHD medication.

## Results

### Patient disposition and baseline characteristics

Patient disposition and baseline characteristics for the overall study population have been fully described previously and were similar across the three treatment groups (Table 1).^10^ A total of 336 patients were enrolled in ten European countries and the full analysis set comprised 317 patients (LDX, n=104; placebo, n=106; OROS-MPH, n=107). The study was completed by 196 individuals (LDX, n=80; placebo, n=42; OROS-MPH, n=74). Table 2 lists the medications previously used to treat ADHD in any treatment group and the numbers of patients that received each medication. The most common previous medication was methylphenidate, which had been used by 158 of the 182 previously treated patients included in the safety population (86.8%). Patients who had not previously received any of the medications listed in Table 2 were considered treatment-naïve.

### Efficacy: Any previous ADHD medication group

**Figure 1:**
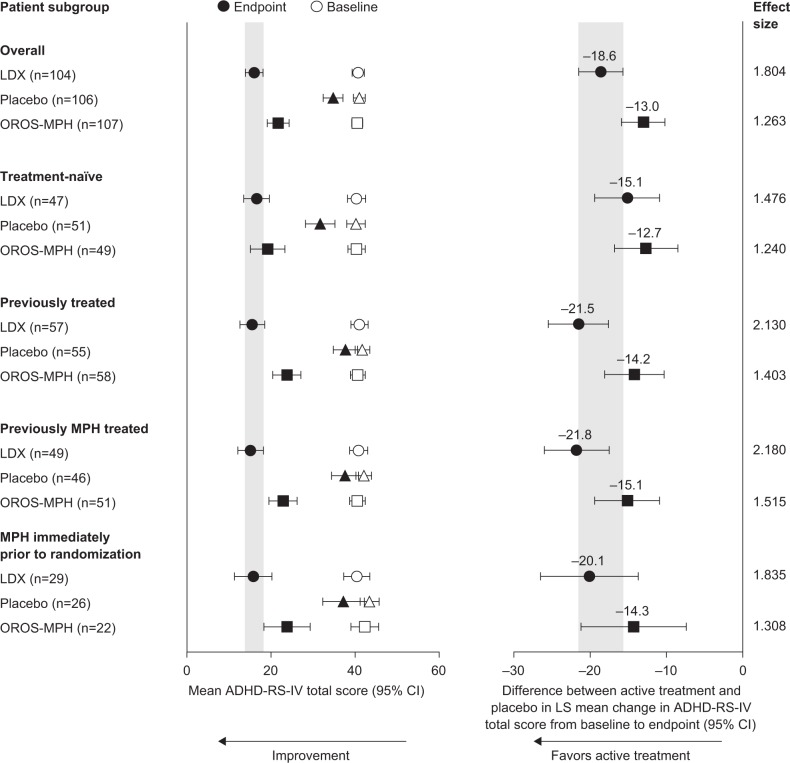
Change in ADHD-RS-IV total scores from baseline to endpoint in treatment-naïve and previously treated patients (full analysis set). **Notes:** ADHD-RS-IV total scores are shown as mean values ±95% CIs. In the left hand panel, open symbols indicate mean baseline scores and closed symbols indicate mean endpoint scores (LDX, circles; placebo, triangles; OROS-MPH, squares). The difference (active drug minus placebo) in LS mean change (±95% CI) from baseline to endpoint is also shown (LDX, circles; OROS-MPH, squares). Gray shading on the left hand panel indicates the 95% CI for the mean ADHD-RS-IV total score at endpoint in the LDX group in the overall study population. On the right hand panel, gray shading indicates the 95% CI for the difference (LDX minus placebo) in LS mean change from baseline to endpoint in the overall study population. Endpoint is the last on-therapy assessment visit with a valid ADHD-RS-IV total score. Immediately prior to randomization is defined as up to 30 days prior to randomization. **Abbreviations:** ADHD, attention-deficit/hyperactivity disorder; ADHD-RS-IV, ADHD rating scale IV; CI, confidence interval; LDX, lisdexamfetamine dimesylate; LS, least-squares; MPH, methylphenidate; OROS-MPH, osmotic-release oral system MPH.

Mean ADHD-RS-IV total scores at baseline were similar in treatment-naïve patients and those who had previously received any ADHD medication. In all subgroups of patients, mean ADHD-RS-IV total scores decreased from baseline to endpoint (Figure 1). Based on 95% confidence intervals, the difference between active treatment (LDX or OROS-MPH) and placebo in least-squares mean change from baseline to endpoint in ADHD-RS-IV total score did not differ from that of the overall study population in both treatment-naïve patients and those who had previously received any ADHD medication (Figure 1). Effect sizes indicated robust treatment effects in all previous treatment subgroups. However, effect sizes were numerically larger in previously treated patients than in treatment-naïve individuals (Figure 1).

Compared with placebo, there were also a greater proportion of patients with an improved CGI-I score (CGI-I score of 1 or 2) at endpoint in the LDX treatment group and in the OROS-MPH reference arm, in both treatment-naïve and previously treated patients (Table 3). The differences between active drug and placebo in the percentage of improved patients at endpoint in each subgroup did not differ from the overall study population.

### Previous MPH treatment group

Mean (standard deviation) ADHD-RS-IV total scores at baseline were similar across treatment groups in treatment-naïve patients and in those who had previously received MPH, either at any time or immediately prior to randomization (Figure 1). The differences (active drug minus placebo) in least-squares mean change from baseline to endpoint in ADHD-RS-IV total scores in patients previously treated with MPH, either at any time or immediately prior to randomization, were similar to those of the overall study population based on 95% confidence intervals (Figure 1). Effect sizes indicated robust treatment effects in previously MPH-treated patients and, like patients who had previously received any ADHD medication, effect sizes in these subgroups were numerically larger than in treatment-naïve individuals.

### Effect of previous treatment on optimal doses of LDX and OROS-MPH

The different previously treated subgroups each received similar mean (standard deviation) doses of LDX (overall 46.1 [11.87] mg/day, n=104; treatment-naïve 44.4 [11.65] mg/day, n=47; previously treated 47.5 [11.96] mg/day, n=57; previously MPH treated 47.1 [12.15] mg/day, n=49; MPH immediately prior to randomization 49.2 [10.70] mg/day, n=29) or OROS-MPH (overall 37.5 [10.06] mg/day, n=107; treatment-naïve 37.0 [9.81] mg/day, n=49; previously treated 37.9 [10.32] mg/day, n=58; previously MPH treated 38.5 [10.13] mg/day, n=51; MPH immediately prior to randomization 38.7 [10.14] mg/day, n=22).

## Discussion

Establishing the efficacy of LDX among previously treated patients is of clinical relevance in Europe, where LDX is approved for use for patients with ADHD who have responded inadequately to MPH. In this randomized, controlled trial, approximately half (54.8%) of patients in the study had received previous ADHD medication and, of these, the majority (158/182 patients; 86.8%) had received MPH. The present post hoc analyses indicated that the response to LDX treatment was similar in subgroups of patients categorized according to whether or not they had previously received ADHD medication, and was similar to that observed for the overall study population.

Several lines of evidence indicate that the outcome of treatment with one ADHD medication does not predict the success of treatment with a second.^16^ The Multimodal Treatment Study of Children with ADHD (MTA) examined potential moderators of treatment response and concluded that previous medication did not predict future response to medication.^17^,^18^ In addition, crossover trials have demonstrated differential clinical responses for individuals treated sequentially with MPH and amphetamines or vice versa.^19^,^20^ The present findings are also consistent with a previous post hoc analysis of a US-based, randomized, double-blind study that indicated that the efficacy of LDX treatment was similar in the subgroup of patients who were receiving MPH treatment at study entry and the overall study population.^21^ Finally, a head-to-head, randomized, double-blind study comparing LDX and atomoxetine demonstrated a robust LDX treatment effect among patients who had previously responded inadequately to MPH.^22^

In the present study, the response to LDX was robust in all subgroups, as indicated by the large effect sizes for the reduction in ADHD-RS-IV total score. However, effect sizes were larger in previously treated patients than among treatment-naïve individuals. Analysis of ten randomized, placebo-controlled trials assessing the efficacy of the nonstimulant ADHD medication atomoxetine in children indicated that a placebo response was more likely in treatment-naïve children than in patients who had previously been treated with ADHD medication.^23^ In the present analysis, the response to placebo was also numerically greater in the treatment-naïve subgroup than in previously treated patients (for both ADHD-RS-IV and CGI-I assessments) and this is likely to have contributed to the smaller effect size in the treatment-naïve subgroup.

The OROS-MPH active reference arm was included in SPD489-325 to provide internal validation of the study design and to contextualize results. A previous post hoc analysis compared the relative benefits of LDX and OROS-MPH treatment in the overall SPD489-325 study population, finding that LDX was significantly more effective than OROS-MPH on several treatment outcomes.^12^ However, the conclusions of this analysis should be considered in light of its post hoc nature.^12^ It should also be noted that the maximum permitted dose of OROS-MPH in Europe is 54 mg/day, which is lower than the 72 mg/day permitted in other regions and which may have limited its efficacy.^12^ The current analysis makes no attempt to compare the relative benefits of LDX and OROS-MPH in patient subgroups because the patient numbers involved were too small to support inferential statistics.

Because the study excluded patients who were well controlled on their current medication with acceptable tolerability, it is noteworthy that OROS-MPH was similarly effective in the overall study population and in patients who had previously received MPH therapy. One possible explanation for this is that the dosage of previous treatment was suboptimal, whereas in the present study both active treatments were dose-optimized. A second possibility is that the formulation of MPH may have differed between the study drug, OROS-MPH, and previous MPH treatment. For example, patients may previously have received short-acting MPH which has been associated with lower levels of treatment adherence than once-daily, long-acting formulations such as OROS-MPH.^24^ Finally, it was evident in the MTA study that an MPH treatment regimen delivered within a controlled clinical trial setting was significantly more effective than treatment as usual within a community care setting, even though most patients undergoing community care were treated with MPH,^18^ indicating that the care and support provided in a clinical trial setting is likely to produce enhanced treatment benefits compared with the same treatment in the absence of such support. Further interpretation of this result is limited owing to the lack of information about why previous medications were discontinued.

Full safety outcomes from this study have been published previously^10^,^12^ and were not examined further in the present post hoc analyses. In summary, the proportion of patients reporting treatment emergent adverse events was 72.1% in the LDX treatment group, 57.3% in the placebo group, and 64.9% in the OROS-MPH group. Low proportions of patients reported serious treatment emergent adverse events across all treatment groups (LDX, 2.7%; placebo, 2.7%; OROS-MPH, 1.8%) and few patients experienced treatment emergent adverse events leading to discontinuation (LDX, 4.5%; placebo, 3.6%; OROS-MPH, 1.8%).

When interpreting the LDX treatment response in these analyses, it should be noted that study SPD489-325 was not powered to detect significant effects between subgroups and, although the subgroups examined here were based on prerandomization patient characteristics, they were not prespecified. In addition, it is unclear whether the specific inclusion and exclusion criteria relating to previous medication response/exposure would have impacted on the LDX treatment response. The short-term nature of this study and the exclusion of patients with comorbidities may limit the conclusions to be drawn from the present findings. Finally, the fact that safety outcomes were not assessed in the previously treated subgroups is another limitation of the present study.

In conclusion, these post hoc analyses of a randomized, double-blind, placebo-controlled trial, which enrolled a large number of patients at multiple centers across Europe, support the use of LDX as an effective therapy for treatment-naïve children and adolescents with ADHD as well as those who have received previous ADHD medication.

## Figures and Tables

**Table 1:** Baseline demographics (safety population)^a^

Characteristic	LDX (n=111)	Placebo (n=110)	OROS-MPH (n=111)
Age, years, mean (SD)	10.9 (2.9)	11.0 (2.8)	10.9 (2.6)
Sex, male, n (%)	87 (78.4)	91 (82.7)	90 (81.1)
Race, white, n (%)	107 (96.4)	108 (98.2)	107 (96.4)
BMI, kg/m^2^, mean (SD)	19.3 (3.7)	19.0 (3.3)	19.1 (3.2)
Baseline ADHD-RS-IV total score, mean (SD)^b^	41.0 (7.3)	41.2 (7.2)	40.4 (6.8)
Baseline CGI-S rating, mean (SD)^b^	5.0 (0.8)	4.9 (0.8)	5.0 (0.8)
ADHD subtype, n (%)^c^			
Predominantly inattentive	23 (20.7)	16 (14.5)	14 (12.7)
Predominantly hyperactive-impulsive	2 (1.8)	7 (6.4)	1 (0.9)
Combined	86 (77.5)	87 (79.1)	95 (86.4)
Time since ADHD diagnosis, years^c^			
Mean (SD)	2.4 (2.9)	2.2 (2.6)	2.2 (2.5)
Median (range)	1.3 (0.0–10.9)	0.90 (0.0–10.2)	0.8 (0.0–9.0)

**Notes:** Adapted from Coghill D, Banaschewski T, Lecendreux M, et al. European, randomized, phase 3 study of lisdexamfetamine dimesylate in children and adolescents with attention-deficit/hyperactivity disorder. *Eur Neuropsychopharmacol*. 2013;23(10):1208–1218.^10^

a Demographic and baseline characteristics have previously been reported in detail;

b five patients had no baseline ADHD-RS-IV total score or CGI-S rating;

c one patient in the OROS-MPH group was not evaluated for ADHD subtype or time since ADHD diagnosis; percentages are based on the number of patients in each treatment group.

**Abbreviations:** ADHD, attention-deficit/hyperactivity disorder; ADHD-RS-IV, ADHD Rating Scale IV; BMI, body mass index; CGI-S, Clinical Global Impressions-Severity; LDX, lisdexamfetamine dimesylate; OROS-MPH, osmotic-release oral system methylphenidate; SD, standard deviation.

**Table 2:** Summary of lifetime ADHD medications (safety population)

	LDX (n=111) n (%)	Placebo (n=110) n (%)	OROS-MPH (n=111) n (%)	Total (n=332) n (%)
Any selected ADHD medication	64 (57.7)	58 (52.7)	60 (54.1)	182 (54.8)
Methylphenidate or methylphenidate hydrochloride	56 (50.5)	49 (44.5)	53 (47.7)	158 (47.6)
Atomoxetine or atomoxetine hydrochloride	17 (15.3)	15 (13.6)	10 (9.0)	42 (12.7)
Risperidone	7 (6.3)	3 (2.7)	6 (5.4)	16 (4.8)
Amphetamine sulfate	3 (2.7)	7 (6.4)	5 (4.5)	15 (4.5)
Carbamazepine	1 (0.9)	1 (0.9)	2 (1.8)	4 (1.2)
Imipramine or imipramine hydrochloride	1 (0.9)	1 (0.9)	1 (0.9)	3 (0.9)
Tiapride hydrochloride	2 (1.8)	1 (0.9)	0 (0.0)	3 (0.9)
Chloroprothixene or chloroprothixene hydrochloride	1 (0.9)	0 (0.0)	1 (0.9)	2 (0.6)
Piracetam	1 (0.9)	0 (0.0)	1 (0.9)	2 (0.6)
Valproate sodium or valproic acid	1 (0.9)	0 (0.0)	1 (0.9)	2 (0.6)
Amitriptyline	0 (0.0)	0 (0.0)	1 (0.9)	1 (0.3)
Clomipramine	1 (0.9)	0 (0.0)	0 (0.0)	1 (0.3)
Cyroheptadine	1 (0.9)	0 (0.0)	0 (0.0)	1 (0.3)
Desipramine	1 (0.9)	0 (0.0)	0 (0.0)	1 (0.3)
Ergenyl chrono	0 (0.0)	0 (0.0)	1 (0.9)	1 (0.3)
Fluvoxamine	0 (0.0)	0 (0.0)	1 (0.9)	1 (0.3)
Haloperidol	0 (0.0)	0 (0.0)	1 (0.9)	1 (0.3)
Lamotrigine	1 (0.9)	0 (0.0)	0 (0.0)	1 (0.3)
Omega-3 fatty acids	1 (0.9)	0 (0.0)	0 (0.0)	1 (0.3)
Perazine	0 (0.0)	0 (0.0)	1 (0.9)	1 (0.3)
Sedariston	0 (0.0)	1 (0.9)	0 (0.0)	1 (0.3)
Sertraline hydrochloride	0 (0.0)	1 (0.9)	0 (0.0)	1 (0.3)
Thioridazine	0 (0.0)	0 (0.0)	1 (0.9)	1 (0.3)
Topiramate	1 (0.9)	0 (0.0)	0 (0.0)	1 (0.3)
Zappelin	0 (0.0)	1 (0.9)	0 (0.0)	1 (0.3)

**Notes:** Patients may have been previously treated with more than one ADHD medication during their lifetime but were only counted once in each drug category. All medication names are based on the terminology provided by the individual study investigators.

**Abbreviations:** ADHD, attention-deficit/hyperactivity disorder; LDX, lisdexamfetamine dimesylate; OROS-MPH, osmotic-release oral system methylphenidate.

**Table 3:** Proportions of improved patients (CGI-I score of 1 or 2) at endpoint (full analysis set)

Patient subgroups	Percentage of improved patients (CGI-I 1 or 2) at endpoint (95% CI)	Difference in percentage of improved patients relative to placebo (95% CI)
**Overall study population (n**=**317)**		
LDX (n=104)	78.0 (69.9, 86.1)	63.6 (53.0, 74.1)
Placebo (n=106)	14.4 (7.7, 21.2)	
OROS-MPH (n=107)	60.6 (51.2, 70.0)	46.2 (34.6, 57.7)
**Patient subgroups based on medication history**		
Treatment-naïve (n=147)		
LDX (n=47)	80.4 (69.0, 91.9)	60.8 (45.0, 76.6)
Placebo (n=51)	19.6 (8.7, 30.5)	
OROS-MPH (n=49)	63.8 (50.1, 77.6)	44.2 (26.7, 61.8)
Previously treated with any ADHD medication (n=170)		
LDX (n=57)	75.9 (64.5, 87.3)	66.5 (52.6, 80.3)
Placebo (n=55)	9.4 (1.6, 17.3)	
OROS-MPH (n=58)	57.9 (45.1, 70.7)	48.5 (33.4, 63.5)

**Note:** Endpoint was defined as the last on-therapy treatment visit with a valid assessment score.

**Abbreviations:** ADHD, attention-deficit/hyperactivity disorder; CGI-I, Clinical Global Impressions-Improvement; CI, confidence interval; LDX, lisdexamfetamine dimesylate; OROS-MPH, osmotic-release oral system methylphenidate.
